# Cervical cancer screening practices among patients with autoimmune and inflammatory rheumatic diseases: a descriptive cross-sectional observational study

**DOI:** 10.1186/s12905-026-04475-2

**Published:** 2026-04-25

**Authors:** Charlotte Delattre, Mayalen Uthurriague, Thomas Barnetche, Marie Lambert, Nadia Mehsen-Cetre, Estibaliz Lazaro, Marie-Elise Truchetet, Claude Hocké, Christophe Richez, Valérie Bernard

**Affiliations:** 1 Department of Gynecology and Reproductive Medicine, CHU de Bordeaux, Bordeaux, F-33000 France; 2 Rheumatology Department, Hôpital Pellegrin, National Reference Center for Systemic Autoimmune Rare Diseases RESO, Bordeaux University Hospital, Bordeaux, France; 3 Department of Internal Medicine, National Reference Center for Systemic Autoimmune Rare Diseases RESO, Hôpital Haut Lévêque, Bordeaux University Hospital, Pessac, France; 4 Bordeaux University, CNRS, Immunoconcept, UMR 5164, 146 rue Léo Saignat, Bordeaux, 33076 France; 5 Univ. Bordeaux, Bordeaux Institute in Oncology-BRIC-BioGo Team, INSERM U1312, Bordeaux, F-33000 France

**Keywords:** Cervical cancer screening, Gynaecological follow-up, Preventive care, Autoimmune inflammatory rheumatic diseases, Cervical cancer screening knowledge

## Abstract

**Background:**

Immunocompromised women with autoimmune and inflammatory rheumatic diseases (AIIRDs) are at increased risk of human papillomavirus (HPV) infection and cervical intraepithelial neoplasia, necessitating appropriate cervical cancer screening (CCS). However, there are still limited reports on CCS practices in patients with AIIRD. We assessed the proportion of women with AIIRDs undergoing CCS in accordance with current European guidelines, and factors associated with appropriate or inappropriate screening.

**Methods:**

We conducted a cross-sectional study at Bordeaux University Hospital (July 2023–July 2024). Women ≥ 18 years with confirmed AIIRDs (systemic lupus erythematosus, systemic sclerosis, Sjögren’s syndrome, Sharp’s syndrome, rheumatoid arthritis, or spondyloarthritis) completed a standardized self-administered questionnaire on rheumatologic and gynaecologic follow-up, CCS practices, and knowledge about HPV/CCS.

**Results:**

Of the 309 participants included, 302 provided the date of their last CCS. Of these, 120 were classified as high risk for cervical pathology (receiving targeted therapies or immunosuppressive treatment), and 182 as lower risk. Overall, 188 of 302 women (62.3%) reported undergoing CCS in accordance with current guidelines. However, among high-risk patients, 45 of 120 (37.5%) had received a cervical smear within the past year. Inadequate screening was significantly associated with a low level of education, more frequent rheumatologic follow-up, and limited knowledge of cervical pathology. Notably, 16% of participants reported not knowing the purpose of the cervical smear.

**Conclusion:**

Women with AIIRDs may benefit from increased CCS awareness among rheumatologists and improved patient education by gynaecologist to promote screening. Further studies are needed to characterise disease- and treatment-specific risks more accurately, establish appropriate screening intervals, and optimise CCS practices for women with AIIRDs.

**Trial registration:**

The study was registered on ClinicalTrials.gov (NCT05961267, date of registration 2023-07-17).

**Supplementary Information:**

The online version contains supplementary material available at 10.1186/s12905-026-04475-2.

## Background

Cervical cancer is primarily caused by persistent infection with high-risk human papillomavirus (HPV), a process that may be facilitated by immunosuppression [1, 2]. The association between immunosuppression and cervical cancer risk is well established in individuals with human immunodeficiency virus (HIV) infection and in organ transplant recipients receiving immunosuppressive therapy. However, the extent of this risk in patients with autoimmune inflammatory rheumatic diseases (AIIRDs) remains uncertain [3]. AIIRD patients often exhibit intrinsic immune system dysfunction and are frequently treated with immunosuppressive agents, both of which may increase susceptibility to infections and malignancies [4, 5]. Although prior studies have not demonstrated an increased incidence of cervical cancer in this population, a higher prevalence of cervical HPV infection has been reported in women with systemic lupus erythematosus (SLE), as well as an elevated risk of cervical intraepithelial neoplasia across various AIIRDs [6–8]. These findings underscore the importance of regular cervical cancer screening (CCS) and adherence to screening programmes in women with AIIRDs. However, several studies have documented suboptimal CCS rates in these patients, which may be attributed to the chronic and disabling nature of their underlying diseases [9–11].

Specific guidelines for CCS in AIIRD patients have been published by various medical societies [12]. In 2016, the European League Against Rheumatism (EULAR) recommended annual Pap smears for women receiving immunosuppressive treatment; however, no specific drugs were identified [13]. The French Society of Colposcopy and Cervical Pathology recommends annual follow-up in immunosuppressed women (unspecified), including a comprehensive examination of the vulva, cervix, and vagina, accompanied by cytological analysis (with or without HPV testing), regardless of age. In addition, French SLE experts recommend annual Pap smears for immunocompromised women with SLE, including those on biotherapy or immunosuppressive agents, while advising screening in line with general population guidelines for those not receiving immunosuppressive therapy [14].

We assessed the CCS rate in accordance with 2016 European guidelines among women with AIIRD, including SLE, systemic sclerosis (SS), Sharp’s syndrome, Sjögren’s syndrome (SjS), rheumatoid arthritis (RA), and spondyloarthritis (SpA). Furthermore, we investigated factors associated with appropriate or inappropriate screening, with a particular focus on patients’ knowledge of HPV and CCS.

## Methods

### Study design

A descriptive cross-sectional observational study was conducted at Bordeaux University Hospital, in collaboration with the Reference Centre for Autoimmune and Autoinflammatory Rare Diseases between July 2023 and July 2024.

### Patients

We included women aged ≥ 18 years with a confirmed diagnosis of SLE, SS, SjS, Sharp’s syndrome, RA, or SpA. Eligible participants were required to be French-speaking, free from comprehension disorders, and affiliated with or benefiting from a social security scheme. Exclusion criteria included women with no history of sexual intercourse, those who had undergone total hysterectomy, and individuals referenced in Articles L 1121-5 to L 1121-8 of the French Public Health Code (persons deprived of liberty by judicial or administrative decision, adults under legal protection, or individuals unable to provide informed consent).

### Technical information

Women were enrolled either during routine follow-up (consultation or hospitalisation) or were invited to participate by post. A standardised self-administered questionnaire was specifically developed for this study and was comprised of four sections (Supplemental Fig. 1). The first section gathered general demographic and social information. The second section focused on rheumatic disease characteristics, treatment, and follow-up. Treatments were categorised as disease-modifying anti-rheumatic drugs (DMARDs), including conventional synthetic DMARDs (csDMARDs), biological DMARDs (bDMARD), and targeted synthetic DMARDs (tsDMARDs), as well as other immunosuppressive drugs and glucocorticoids. The third section collected data on gynaecological follow-up. The fourth section addressed CCS practices and assessed patient knowledge regarding HPV and CCS. When feasible, self-reported CCS information was cross-checked against the hospital electronic medical record for participants followed at Bordeaux University Hospital. The patient’s record was systematically recorded in the computer. All collected data were pseudo-anonymised to ensure confidentiality and protect participant privacy prior to being entered into a secure electronic database.

### Outcomes

The primary outcome was the proportion of women who had undergone adequate CCS in accordance with current European rheumatology guidelines. Patients were considered at high risk of cervical pathology (requiring annual CCS) if they were currently receiving targeted therapies, including bDMARD, tsDMARD, or other immunosuppressive treatments including corticosteroids. Low-risk patients (currently treated with csDMARDs or not receiving treatment) were considered adequately screened if they had a Pap smear or HPV test within the past three years, in line with French guidelines at the time. Indeed, several systematic reviews and observational studies have shown that biological agents (bDMARDs) and targeted synthetic (tsDMARDs) exhibit a more pronounced immunosuppressive profile than conventional synthetic DMARDs (csDMARDs), particularly regarding the risk of serious infections [15–17]. The classification of patients as “low-risk” versus “high risk” represents our operational definition, based on our own interpretation of the available guidelines. Since HPV testing had only been routinely available in France for three years at the time of MARIGYN inclusion, we did not distinguish between Pap smear and HPV testing in the results and refer to them collectively as CCS.

Secondary outcomes included rheumatological and gynaecological follow-up, HPV vaccination status, the proportion of patients with a history of abnormal cervical cytology, patients’ knowledge of cervical health, and factors associated with appropriate CCS.

### Statistical analyses

Patient characteristics and clinical outcomes were reported as mean (with standard deviation) or median (with range) for continuous variables, and as frequency (percentage) for categorical variables. Quantitative variables were compared using the Student’s *t*-test, and categorical variables using the Chi-square test or Fisher’s exact test, as appropriate. We assessed multicollinearity before interpreting the multivariable logistic regression model by calculating variance inflation factors using a linear model including the same explanatory variables. Statistical analyses were performed using STATA software (version 13.1; StataCorp, College Station, Texas, USA). P-values < 0.05 were considered statistically significant.

### Ethical approval and consent to participate

The MARIGYN project received approval from the French Ethics Committee (Comité de Protection des Personnes Ile de France III) and was conducted in accordance with the ethical principles of the Declaration of Helsinki and Good Clinical Practice guidelines. The study was registered on ClinicalTrials.gov (NCT05961267, date of registration 2023-07-17) and in the European database (ID-RCB 2023-A01207-38). All patients provided written informed consent before participating.

## Results

**Fig. 1:**
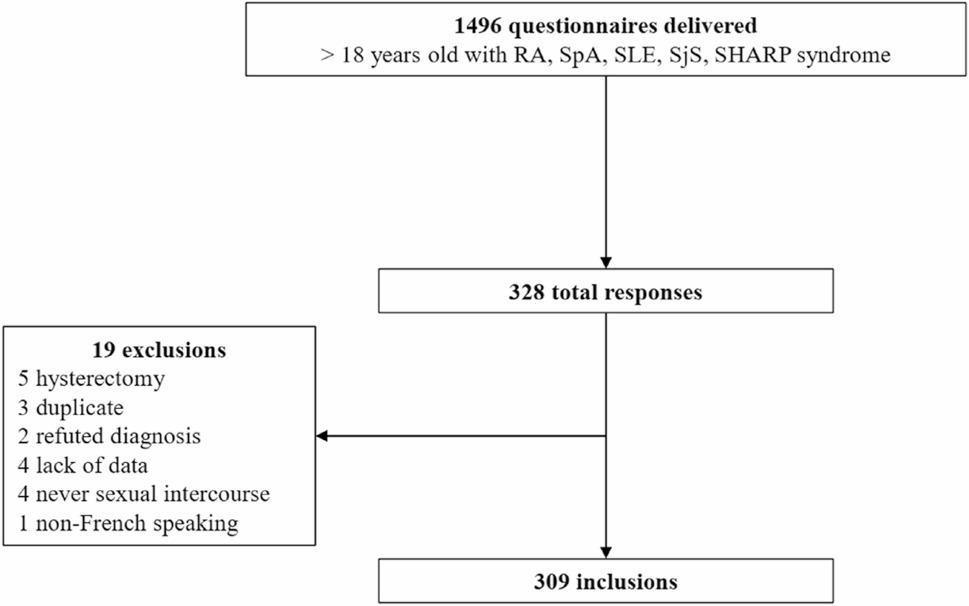
Flow chart. RA, rheumatoid arthritis; SpA, spondyloartritis; SLE, systemic lupus erythematosus; SS, systemic sclerosis; SjS, Sjogren’s syndrome

The survey was distributed to 1,496 eligible patients from the rheumatology department at Bordeaux University Hospital. In total, 309 AIIRD patients were ultimately included in the analysis (Fig. 1). Table 1 presents the baseline population characteristics. The median age was 47.5 years. Over half of the participants (51.3%) had a high level of education. Among all AIIRDs, SLE was the most prevalent, affecting 134 patients (43.4%). The most commonly prescribed treatments were csDMARDs, with hydroxychloroquine prescribed in 125 cases (40.5%). Overall, 167 patients (54.1%) received at least one csDMARD, 97 patients (31.4%) were treated with a targeted therapy, and 29 patients (9.4%) received an immunosuppressive agent. Targeted therapies were predominantly prescribed for patients with RA and SpA (84.8%), with anti-tumour necrosis factor agents being the most frequent. Immunosuppressive therapies were used in patients with systemic autoimmune diseases. Glucocorticoids were prescribed for 75 patients (24.3%), whereas 45 patients (14.6%) were not receiving any treatment. Regarding rheumatological follow-up, 250 patients (80.9%) had at least one specialist consultation per year, with 98 patients (32.1%) attending more frequently than every 6 months.

**Table 1:** Population characteristics

Characteristics	*N* = 309
General characteristics	
Age (years), mean (range)	47.5 (21–81)
Age at diagnosis (years), mean ± SD	34 ± 14.4
Menopausal, n (%)	143/309 (46.3%)
BMI (kg/m²), mean ± SD	23.1 ± 5.8
Education level, n (%)	
No degree	20/302 (6.6%)
Less than high school diploma	58/302 (19.2%)
High school diploma	69/302 (22.9%)
University degree	155/302 (51.3%)
Family status, n (%)	
Single	70/305 (23%)
Couple	235/305 (77%)
AIIRD, n (%)	
RA	46/309 (14.9%)
SpA	35/309 (11.3%)
SLE	134/309 (43.4%)
SS	59/309 (19.1%)
SjS	31/309 (10%)
Sharp’s syndrome	4/309 (1.3%)
Treatments, n (%)	
csDMARD	167/309 (54.1%)
Hydroxychloroquine	125 (40.5%)
Methotrexate	52 (16.8%)
Salazopyrine	2 (0.7%)
Leflunomide	2 (0.7%)
Targeted therapy (bDMARD or tsDMARD)	97/309 (31.4%)
TNFi	41 (13.3%)
Anti-IL-6	16 (5.2%)
Orencia	14 (4.5%)
Anti-CD20	12 (3.9%)
Belimumab	10 (3.2%)
JAK inhibitor	4 (1.3%)
Anti-IL-17	1 (0.3%)
Other immunosuppressive therapy	29/309 (9.4%)
Mycophenolate mofetil	17 (5.5%)
Azathioprine	10 (3.2%)
Calcineurin inhibitor	2 (0.7%)
Corticosteroids	75 (24.3%)
No treatment	45 (14.6%)
Rheumatological follow-up, n (%)	
Frequency	
Less than once a year	55/305 (18%)
1–2 times a year	152/305 (49.8%)
> 2 times a year	98/305 (32.1%)
Influenza vaccination up to date, n (%)	139/304 (45.7%)

*SD* Standard deviation, *BMI* Body mass index, *AIIRDs* Autoimmune and inflammatory rheumatic diseases, *RA* Rheumatoid arthritis, *SpA* Spondyloarthritis, *SLE* Systemic lupus erythematosus, *SS* Systemic sclerosis, *SjS* Sjögren’s syndrome, *csDMARD* Conventional synthetic disease-modifying antirheumatic drug, *bDMARD* Biologic DMARD, *tsDMARD* targeted synthetic DMARD, *TNFi T*umour necrosis factor inhibitor, *IL* Interleukin

Table 2 presents gynaecological follow-up and characteristics related to cervical cancer. Regarding gynaecological care, the majority of patients (80.5%) reported having at least one gynaecologist, and 81.1% expressed satisfaction with their follow-up. Overall, 164 patients (54.8%) stated that they had not been referred to a gynaecological health professional by their specialist physician. Among those who had not consulted a gynaecologist despite their physician’s recommendation, the most commonly cited reason was the unavailability of a gynaecologist in their vicinity. In terms of cervical cancer risk factors, 46 patients (15.1%) were current smokers, 194 (66.7%) reported having had three or more sexual partners, 64 (21.8%) had a history of sexually transmitted infection (STI), and 161 (52.6%) were multiparous. Of the 99 patients eligible for HPV vaccination, 23 (23.2%) reported having received it. Based on self-reported data and consistent data selection, 43 patients (13.2%) had undergone colposcopy following an abnormal smear, and 21 (6.8%) reported a history of cervical conisation.

**Table 2:** Gynaecological follow-up and characteristics of cervical pathology

Gynaecological follow-up	
Professional gynaecological follow-up, n (%)	
At least one gynaecologist	240/298 (80.5%)
Midwives only	21/298 (7.1%)
General practitioner only	20/298 (6.7%)
No follow-up	17/298 (5.7%)
Frequency of gynaecological visits, n (%)	
Last visit within 1 year	194/297 (65.3%)
Last visit 2–3 years ago	71/297 (23.9%)
Less than once every 3 years or never	32/297 (10.8%)
Satisfaction with follow-up, n (%)	
Yes	236/291 (81.1%)
No or unknown	55/291 (18.9%)
Referral to gynaecologist by specialist physician, n (%)	
Yes	135/299 (45.2%)
No or unknown	164/299 (54.8%)

Risk factors for cervical cancer	
Smoking status, n (%)	
Current smoker	46/305 (15.1%)
Ex-smoker	104/305 (34.1%)
Never smoked	155/305 (50.8%)
Number of sexual partners, n (%)	
1	66/300 (22.0%)
2	40/300 (13.3%)
≥ 3	194/300 (66.7%)
History of STI, n (%)	64/294 (21.8%)
Multiparity, n (%)	161/306 (52.6%)
Current use of COC, n (%)	7/209 (2.3%)

HPV vaccination history	
Women potentially eligible for HPV vaccination, n (%)	
At least 1 dose received	29/99 (29.3%)
Complete vaccination (≥ 2 doses)	23/99 (23.2%)
Reason for non-vaccination, n (%)	
Lack of knowledge about vaccine availability	57/69 (82.6%)
Concern about vaccine safety	6/69 (8.7%)
Perceived contraindication	1/69 (1.5%)
Other reasons	5/69 (7.2%)

History of cervical pathology, n (%)	
Colposcopy for abnormal smear	43/309 (13.9%)
CIN	31/309 (10%)
Cervical conization (for CIN 1, 2, or 3)	21/309 (6.8%)

*STI* Sexually transmitted infection, *COC* Combined oral contraceptive, *HPV* Human papillomavirus, *CIN* cervical intraepithelial neoplasia

Table 3 summarises participants’ knowledge about HPV and CCS. In total, 50 patients (16.2%) reported not knowing the purpose of CCS, and 237 (80.9%) stated they had not been informed about the risks associated with HPV. Regarding HPV vaccine protection, 40 patients (13.7%) were unsure whether vaccination exempted them from the need for cervical screening.

**Table 3:** Knowledge about HPV and CCS

HPV risk information provided by a specialist physician, *n* (%)	
No	237/293 (80.9%)
Yes	56/293 (19.1%)
Perceived utility of cervical smear*, n (%)	
CCS	259/309 (83.8%)
STI screening	121/309 (39.2%)
Vaginal flora assessment	69/309 (22.3%)
Unknown reason	20/309 (6.5%)
Other	4/309 (1.3%)
Perception of CCS necessity despite HPV vaccination, n (%)	
Yes	250/292 (85.6%)
No	2/292 (0.7%)
Unknown	40/292 (13.7%)
CCS frequency, n (%)	
Every year	92/297 (31%)
Every 2–3 years	150/297 (50.5%)
Every 5 years	22/297 (7.4%)
Unknown	33/297 (11.1%)

*HPV* Human papillomavirus, *CCS* Cervical cancer screening, *STI* Sexually transmitted infection

^*^Multiple responses allowed

**Fig. 2:**
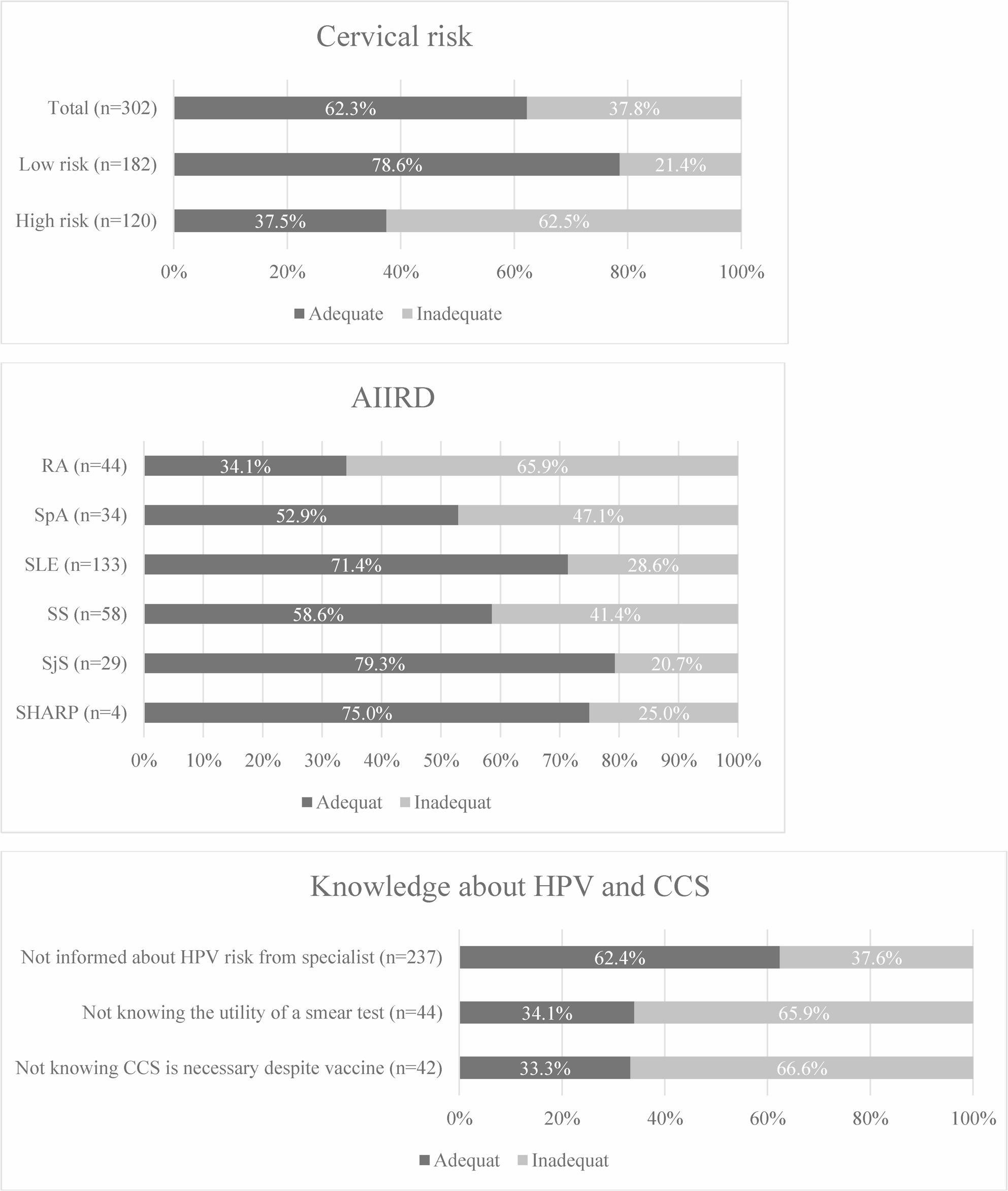
Adequate CCS according to current European guidelines, stratified by cervical cancer risk level, AIIRD subtype, and patient knowledge of HPV and CCS. Low-risk group: Women with AIIRDs who are either untreated or receiving csDMARDs. High-risk group: Women with AIIRDs treated with bDMARDs, tsDMARDs, or other immunosuppressive therapies. CCS, cervical cancer screening; AIIRDs, autoimmune and inflammatory rheumatic diseases; RA, rheumatoid arthritis; SpA, spondyloarthritis; SLE, systemic lupus erythematosus; SS, systemic sclerosis; SjS, Sjögren's syndrome; csDMARD, conventional synthetic disease-modifying antirheumatic drug; bDMARD, biologic DMARD; tsDMARD, targeted synthetic DMARD

Figure 2 presents CCS rates according to current European guidelines. Overall, 188 patients (62.3%) were adequately screened. Of them, 182 were classified as low risk and 120 as high risk. Adequate screening was observed in 143 of 182 low-risk patients (78.6%) and in 45 of 120 high-risk patients (37.5%). By disease category, 34.1% of women with RA and 71.4% of those with SLE were adequately screened. Inadequate screening was more frequent among women who were unaware of the purpose and necessity of cervical smears, particularly in the context of HPV vaccination. Adequate CCS was significantly associated with the frequency of gynaecological follow-up and patient satisfaction with that follow-up (both *p* < 10^− 4^). In contrast, inadequate CCS was significantly associated with a lower level of education (*p* = 0.017), more frequent rheumatological follow-up (*p* = 0.001), a higher number of sexual partners, and lack of knowledge regarding the utility and necessity of cervical screening despite HPV vaccination (both *p* < 0.001; Table 4). The multivariate analysis found that a higher frequency of rheumatology follow-up, lower frequency of gynecological follow-up, and not knowing the purpose of a cervical smear were associated with inadequate screening (Tables 5).

**Table 4:** Comparison of adequate and inadequate CCS

Variables	Adequate CCS	Inadequate CCS	*p*-value
Total, n (%)	188 (62.3%)	114 (37.8%)	
General characteristics			
Age (years), mean ± SD	47.8 ± 11.9	49.7 ± 1.6	0.25
Age at diagnosis (years), mean ± SD	34.4 (13.6)	37.4 (15.4)	0.12
Menopausal	84/188 (44.7%)	53/114 (46.5%)	0.76
BMI (kg/m²), mean ± SD	24.3 ± 5.1	24.7 ± 6.8	0.63
Low education level (no degree), n (%)	6 (3.2%)	11 (9.9%)	0.017
Rheumatology follow-up, n (%)			
Frequency			0.001
Less than once a year	42/188 (22.3%)	13/111 (11.7%)	
1–2 times per year	100/188 (53.2%)	49/111 (44.1%)	
> 2 times a year	46/188 (24.5%)	49/111 (44.1%)	
Flu vaccine up to date	74/188 (39.4%)	61/110 (55.5%)	0.007
Referral to gynaecologist	78 (42.2%)	54/107 (50.5%)	0.17
Gynaecological follow-up, n (%)			
Frequency			< 0.001
At least once a year	137/184 (74.5%)	52/106 (49.1%)	
Every 2–3 years	42/184 (22.8%)	28/106 (17.5%)	
Less than every 3 years	5/184 (2.7%)	26/106 (24.53%)	
No gynaecological provider	0/179 (0%)	5/101 (5%)	0.007
Satisfied with follow-up	161/184 (87.5%)	68/100 (68%)	< 0.001
Risk factors for cervical cancer, n (%)			
Smoking status, n (%)	26 (14%)	20 (17.9%)	0.369
Current use of COC	2/188 (1.1%)	5/114 (4.4%)	0,063
First sexual intercourse at a young age	67/188 (35.6%)	41/111 (36.9%)	0.82
3 or more sexual partners	129/186 (69.4%)	64/111 (57.7%)	0.041
History of STI	45/184 (24.5%)	19/107 (17.8%)	0.18
Multiparity	97/188 (51.6%)	58/111 (52.3%)	0.91
Knowledge about HPV and CCS, n (%)			
Does not know purpose of smear test	15/188 (8%)	29/114 (25.4%)	< 0.001
Unaware CCS is needed despite HPV vaccination	14/184 (7.6%)	28/106 (26.4%)	< 0.001
Not informed about HPV risk by specialist	148/184 (80.4%)	89/108 (82.4%)	0.68

*CCS* Cervical cancer screening, *SD* Standard deviation, *BMI* Body mass index, *COC* Combined oral contraceptive, *HPV* Human papillomavirus, *STI* Sexually transmitted infection

**Table 5:** Multivariate analysis of factors associated with inadequate CCS

Variable	OR [95%]	*p*-value
Education level		
University degree	Ref	
High school diploma	0.49 [0.11–2.20]	0.35
Less than high scool diploma	0.43 [0.10–1.92]	0.27
No degree	0.50 [0.12–2.1]	0.33
Rheumatology follow-up, n (%)		
Frequency		
Less than once a year	Ref	
1–2 times per year	1.96 [0.84–4.55]	0.12
> 2 times a year	6.1 [2.5–14.9]	< 0.001
Gynaecological follow-up, n (%)		
Frequency		
At least once a year	Ref	
Every 2–3 years	2.13 [1.13-4.00]	0.02
Less than every 3 years	13.3 [4.22–41.7]	< 0.001
Age	0.99 [0.97–1.02]	0.56
Does not know purpose of cervical smear	2.6 [1.12–5.91]	0.02

## Discussion

Our study evaluated the proportion of women with AIIRDs undergoing CCS in accordance with European guidelines and identified associated factors. Only 62.3% of participants were screened in line with EULAR recommendations, emphasising a substantial gap between guideline-based recommendations and real-world clinical practice in France. CCS rates varied according to disease type and country. Among patients with SLE, the screening rate (71.4%) was comparable to that reported in cohorts from the United States (54–73.4%). In contrast, the rate among patients with RA was significantly lower (34.1%) than that observed in the United States and the United Kingdom (70–83%) [10, 18]. This discrepancy may be attributed to the lack of consensus regarding screening frequency. Within the European Union, EULAR recommends annual cervical smears for patients receiving immunosuppressive therapy; however, no specific medications are identified. In contrast, guidelines from the United States and the United Kingdom (ASCCP) advise initiating annual screening from the age of 21, with the interval extended to every 3 years if results remain normal [13, 19]. Considering the scarcity of studies addressing AIIRD patients, screening recommendations are often extrapolated from data on women living with HIV, for whom the natural history of cervical cancer is better characterised through prospective studies [20–22]. Recent studies have evaluated the role of HPV testing and have suggested extending the interval between cervical smear tests in HIV patients following a negative result [23–25]. Since 2020, the wider implementation of HPV testing may allow for longer screening intervals, similar to those recommended for women with HIV. This less restrictive approach could enhance adherence to screening protocols. In our study, 79.9% of participants underwent CCS within a 3-year interval (Supplementary Fig. 2); this rate is higher than that observed in the general population in France and aligns with findings from other studies. The elevated screening rate in our cohort may be attributable to a relatively high socioeconomic status, which correlates with greater participation in CCS programmes [26–28]. It is worth noting that in cases of refusal of a gynecological examination or in specific situations, such as women with reduced mobility, HPV testing can be performed using self-sampling [29].

CCS rate can be influenced by institutional factors and physician- and patient-related characteristics. Compared to studies from the United States, institutional barriers are less pronounced in France, where screening is covered by national health insurance [30]. In our study, a low educational level was as significantly associated with inadequate CCS, consistent with prior studies [31]. Patients with RA were the most inadequately screened among the AIIRD cohort. This may be attributed to the prevalent use of targeted therapies in this group, which places them in a high-risk category for cervical pathology. In our study, high-risk status implied a more stringent screening schedule, with annual CCS required to be considered guideline-concordant. Therefore, the lower “adequate CCS” rate observed in RA may partly reflect this shorter recommended interval rather than a complete lack of screening. However, most of these patients were receiving newer targeted agents, the long-term risk profiles of which remain unclear [32–34]. Disease activity and functional impairment may also contribute to reduced uptake (competing care priorities/healthcare fatigue); however, disease activity scores and disability measures were not collected and could not be evaluated. Notably, specific gynaecological guidelines for CCS in immunosuppressed patients remain limited.

In our study, a high frequency of annual specialist consultations was associated with inadequate CCS. This counterintuitive finding should be interpreted cautiously and may reflect residual confounding: more frequent rheumatology visits could be a proxy for greater disease severity, functional limitation, comorbidity, or more intensive immunosuppressive treatment (and thus classification in a high-risk group requiring a more stringent annual screening interval). Alternatively, frequent specialist follow-up may be associated with fragmented care pathways in which preventive care is deprioritised. Although prior studies have suggested that increased specialist involvement enhances adherence to preventive care, particularly in populations with historically reduced healthcare engagement, this inverse association may reflect a phenomenon of healthcare fatigue among women requiring frequent medical visits. Such fatigue could lead to deprioritisation of primary care and routine screening [30, 35, 36]. This hypothesis is supported by Bruera et al. and further substantiated by the significant association observed between up-to-date influenza vaccination and inadequate CCS. Women receiving influenza vaccination were predominantly those undergoing targeted or immunosuppressive therapies, which may reflect higher disease activity and healthcare utilisation [37, 38].

A key finding of our study is that limited knowledge was strongly associated with inadequate CCS. Inadequate screening was associated with a lack of awareness regarding HPV-related risks and a poor understanding of the purpose of cervical smears. Many patients misinterpreted the test, frequently confusing it with screening for STIs or vaginal flora analysis, indicating an overestimation of their knowledge; this gap is particularly striking given that most participants were under gynaecological care and reported satisfaction with it, emphasising the critical role of gynaecologists in patient education. To our knowledge, this is the first study to investigate this issue in women with AIIRD.

Regarding primary prevention through vaccination, we observed that 23.2% of eligible patients had completed the full HPV vaccination schedule. In the general French population, coverage has been gradually increasing but remains suboptimal, with complete vaccination rates at age 16 estimated at approximately 24% in 2018 and 41.5% in 2022. Although data on vaccination coverage among patients with AIIRDs are limited, most patients in our cohort were diagnosed after the recommended age for HPV vaccination, indicating that their vaccination rates and barriers are likely similar to those in the general population. Current guidelines recommend HPV vaccination before the age of 16, with catch-up vaccination permitted up to age 26, to achieve an optimal humoral response. However, recent studies have suggested that the vaccine-induced humoral response exceeds that elicited by natural infection, regardless of age [39]. Therefore, HPV vaccination should be discussed prior to the initiation of immunosuppressive therapy, in alignment with broader immunisation recommendations, even for individuals > 26 years [40].

This is the first French study to assess CCS in a large cohort of women with various AIIRDs, including RA, SpA, SLE, SS, SjS, and Sharp’s syndrome. Notably, it provides previously unavailable data, particularly for women with SS and SjS. In addition, it is the first study to propose risk stratification based on the type of immunosuppression associated with AIIRDs, where the concept of immunosuppression remains poorly defined.

Our study has several limitations. First, because of the descriptive cross-sectional design, causal inference cannot be made and observed associations may be subject to residual confounding. Although we performed multivariable analyses to adjust for measured confounders, residual confounding cannot be excluded, particularly for unmeasured factors such as disease activity and functional disability. Second, the study relied largely on self-reported data, which introduces the risk of recall bias, misunderstanding of questions, and inaccurate reporting of cervical smear dates. Self-reporting may also be influenced by social desirability and could overestimate guideline-concordant screening, while misremembered dates may alternatively underestimate adherence; importantly, some participants reported limited understanding of the purpose of CCS, which may have affected the accuracy of responses. However, when feasible, CCS information was cross-checked using electronic medical records for participants followed at our institution. Third, although multiple rheumatic diseases were included, interpretation remains constrained by the limited evidence on risk stratification and screening recommendations for conditions other than RA and SLE. Fourth, selection bias is possible given the response rate relative to the number of questionnaires distributed and the likelihood that responders differed from non-responders (e.g., more health-conscious women or those with better healthcare access), which may limit generalisability. Furthermore, this was a single-centre study, conducted in a university hospital, which may further limit the external validity of our findings. In addition, our cohort included a high proportion of women with higher educational attainment; socioeconomic position was therefore only partly captured, as we did not collect immigration status, ethnic origin, income/economic indicators, and educational level may incompletely reflect social vulnerability. Because lack of health insurance coverage was an exclusion criterion, all included patients had social security coverage at the time of enrolment; therefore, cervical cancer screening uptake may have been overestimated, as the most socioeconomically vulnerable patients were not represented in our study population. Moreover, previous use of immunosuppressive treatments such as cyclophosphamide was not recorded, which could have impacted long-term immunosuppression exposure and HPV-related risk. Finally, we did not collect physician-level variables such as rheumatologist experience, which may influence care coordination and preventive practices.

## Conclusions

CCS in women with AIIRDs remains inadequate in 2024, particularly for those at increased risk of HPV infection and cervical intraepithelial neoplasia. Improved CCS awareness among rheumatologists and enhanced patient education by gynaecologists are essential to support appropriate screening uptake in this population. Notably, the concept of immunosuppression is not well defined in the context of AIIRDs. Further studies are needed to characterise disease- and treatment-specific risks more accurately, establish appropriate screening intervals, and optimise CCS practices for women with AIIRDs.

## Supplementary Information

Supplementary Material 1.

Supplementary Material 2.

## Data Availability

The data that support the findings of this study are available from Thomas Barnetche, but restrictions apply to the availability of these data, which were used under license for the current study, and so are not publicly available. Data are however available from the corresponding author upon reasonable request.

## Abbreviations

AIIRD: Autoimmune inflammatory rheumatic diseases
HPV: High-risk human papillomavirus
CCS: Cervical cancer screening
HIV: Human immunodeficiency virus
SLE: Systemic lupus erythematosus
CIN: Cervical intraepithelial neoplasia
EULAR: European League Against Rheumatism
SFPC: French Society of Colposcopy and Cervical Pathology
SS: Systemic sclerosis
SjS: Sjogren’s syndrome
RA: Rheumatoid arthritis
SpA: Spondyloarthritis
DMARD: Disease Modifying Anti-Rheumatic Drug
csDMARD: Conventional synthetic Disease Modifying Anti-Rheumatic Drug
bDMARD: Biological Disease Modifying Anti-Rheumatic Drug
tsDMARD: Targeted synthetic Disease Modifying Anti-Rheumatic Drug
ID: Immunosuppressive drug
CGT: Glucocorticoids
STI: Sexual transmitted infection
US: United States
UK: United Kingdom
ASCCP: American Society for Colposcopy and Cervical Pathology
