# Supplementary material for: Cervical cancer screening practices among patients with autoimmune and inflammatory rheumatic diseases: a descriptive cross-sectional observational study
Source: BMC Womens Health. 2026 Apr 25;26:362. doi: 10.1186/s12905-026-04475-2 (PMC13371678; doi:10.1186/s12905-026-04475-2)
Supplement: Supplementary file 1 — Supplementary Material 1. [file 12905_2026_4475_MOESM1_ESM.pdf]

Supplemental Figure 1: MARIGYN QUESTIONNAIRE: EVALUATION OF GYNECOLOGICAL FOLLOW-UP FOR PATIENTS WITH AUTOIMMUNE DISEASES OR INFLAMMATORY RHEUMATISM

PART 1: GENERAL INFORMATION

- Age:
- Weight:
- Height:
- Education level:
  - No diploma or primary education certificate
  - Middle school diploma
  - High school diploma (general or professional)
  - Higher education diploma
- Family status:
  - Single
  - In a relationship
- Tobacco consumption:
  - I currently smoke
    - Specify the number of cigarettes smoked per day, week, or month: ………………..
    - Specify the number of years spent smoking: …………………………………………………………………………………
  - I smoked in the past
    - Specify the number of cigarettes you smoked per day, week, or month: ………………..
    - Specify the number of years spent smoking: …………………………………………………………………………………
  - I have never smoked
- Alcohol consumption:
  - Never or very occasionally
  - Weekly
  - Daily

PART 2: REGARDING YOUR RHEUMATIC DISEASE

- What is your disease among:
  - Systemic lupus erythematosus
  - Systemic scleroderma
  - Sjögren's syndrome
  - Sharp syndrome - mixed connective tissue disease
  - Rheumatoid arthritis
  - Spondyloarthritis, psoriatic arthritis
  - Other

- What is/are your treatment(s) among the following list:
  - If subcutaneous or intravenous injection (alphabetical order):

- ❑ Actemra
- ❑ Amgevita
- ❑ Amsparity
- ❑ Benepali
- ❑ Benlysta
- ❑ Biltzima
- ❑ Cosentyx
- ❑ Cimzia
- ❑ Cyclophosphamide
- ❑ Enbrel
- ❑ Endoxan
- ❑ Erelzi
- ❑ Flixabi
- ❑ Halimatoz
- ❑ Hefiya
- ❑ Hulio
- ❑ **Humira**
- ❑ Hyrimoz
- ❑ Idacio
- ❑ Ilaris
- ❑ Imeth
- ❑ Imraldi
- ❑ Inflectra
- ❑ Kevzara
- ❑ Kineret
- ❑ Kromeya
- ❑ Lifmior
- ❑ Mabthera
- ❑ Méthotrexate
- ❑ Metoject
- ❑ Nepexto
- ❑ Nordimet
- ❑ Orencia
- ❑ Prextate
- ❑ Remicade
- ❑ Remsima
- ❑ Ritemvia
- ❑ Roactemra
- ❑ Rituzena
- ❑ Rixathon
- ❑ Riximyo
- ❑ Ruxience
- ❑ Simponi
- ❑ Solymbic
- ❑ Stelara
- ❑ Taltz
- ❑ Tremfya
- ❑ Truxima
- ❑ Zessly
- ❑ Autre : …………………..

  - If treatment by tablet (alphabetical order):

- ❑ Adoport
- ❑ Advagraf
- ❑ Anti-inflammatoires non stéroïdiens *
- ❑ Arava
- ❑ Cellcept
- ❑ Colchicine
- ❑ Conferoport
- ❑ Cortancyl
- ❑ Envarsus
- ❑ Imeth
- ❑ Imurel
- ❑ Jyseleca
- ❑ Modigraf
- ❑ Myfortic
- ❑ Novatrex
- ❑ Olumiant
- ❑ Plaquenil
- ❑ Prednisolone
- ❑ Prednisone
- ❑ Prograf
- ❑ Protopic
- ❑ Rinvoq
- ❑ Salazopyrine
- ❑ Solupred
- ❑ Tacromez
- ❑ Xeljanz
- ❑ Autre : ………………..

* (Aspirine, Aspégic, Diclofénac, Flector, Voltarène, Ibuprofene, Advil, Nurofen, Kétoprofène, Profénid, Kétum...)

- Are you taking a blood thinning treatment? (anticoagulant or antiplatelet therapy, such as Coumadin, Previscan, Sintrom, Xarelto, Eliquis, Kardegic, Aspegic)
  - Yes
  - No
- Have you been vaccinated against the flu during the past year?
  - Yes
  - No
- How often do you see your disease specialist (rheumatologist or internist)?
  - Less than once a year
  - 1 to 2 times a year
  - More than 2 times a year

PART 3: REGARDING YOUR GYNECOLOGICAL HISTORY AND FOLLOW-UP

- Do you currently have any condition(s) associated with your disease among the following?
    - High blood pressure
    - High cholesterol
    - Diabetes
    - Migraine with aura (visual disturbances such as light flashes or visual blurring preceding the migraine attack)
    - Migraine without aura
    - APS (antiphospholipid syndrome)
    - Breast or uterine cancer
    - None
- Have you ever had a venous thrombosis (phlebitis or pulmonary embolism) or arterial thrombosis (stroke or heart attack)?
    - Yes (specify the year) ………………………………………………………
    - No
- In your family, have there been any cases of cardiovascular events among the following?
    - Stroke before age 60 in men or before age 65 in women
    - Myocardial infarction (heart attack) before age 60 in men or before age 65 in women
    - Phlebitis or pulmonary embolism before age 50
    - None
- Do you have children?
    - Yes (specify the number of children) ………………………………………
    - No
-
- Have you ever started a pregnancy/pregnancies?
    - Yes
    - No
- If yes, then answer the following questions:
- If no, then go directly to the next question in bold
    - Did you have difficulty conceiving (delay > 1 year):
        - Yes, requiring assisted reproductive technology
        - Yes, without requiring assisted reproductive technology
        - No
    - Have you ever had unplanned pregnancies?
        - Yes
        - No
    - Have you ever had voluntary termination(s) of pregnancy (abortion)?
        - Yes
        - No
    - Have you ever had ectopic pregnancies?
        - Yes
        - No
    - Have you ever had early miscarriages (1st trimester)?
        - Yes
        - No
    - Have you ever had late miscarriages and/or intrauterine fetal death (2nd, 3rd trimester)?
        - Yes
        - No
    - Have you ever had medical termination(s) of pregnancy?
        - Yes
        - No
    - Have you ever carried pregnancy/pregnancies to term with live birth(s)?
        - Yes

        - No
    - Have you ever had complications during your pregnancy/pregnancies?
        - No
        - Hypertension
        - Gestational diabetes
        - Pre-eclampsia
        - Intrahepatic cholestasis of pregnancy
        - Prematurity (delivery before 37 weeks)
        - Fetal malformations
        - Other (specify which one(s)): ……………………………………………
- Do you think your disease and/or your treatment impacts your fertility?
    - Yes
    - No
- Do you think a pregnancy is compatible with your treatment?
    - Yes
    - No
- Do you think your disease has an impact on your pregnancy plans?
    - Yes
    - No
- Have you given up on a pregnancy plan because of your condition?
    - Yes
    - No
- Have you ever considered stopping your treatments to start a pregnancy?
    - Yes
    - No
- Which healthcare professional performs the majority of your gynecological follow-up?
    - Gynecologist
    - Midwife
    - General practitioner
    - None
- How often do you see this healthcare professional for your gynecological follow-up?
    - Several times a year
    - Once a year
    - Once every 2-3 years
    - Once every 4-5 years
    - Less than once every 5 years
    - I have never had a follow-up
- Are you satisfied with your gynecological follow-up?
    - Yes
    - No
    - I don't know
- Has your rheumatologist or internist recommended that you consult a healthcare professional for your gynecological follow-up?
    - Yes
    - No
    - I don't know
    - If yes, have you consulted this healthcare professional?
        - Yes
        - No
    - If you have not consulted this healthcare professional, what were the reasons (check one or more possible reasons)?
        - I don't have a gynecology practitioner nearby (gynecologist, midwife, or general practitioner)
        - I lack free time to consult
        - I am apprehensive about the gynecological examination due to modesty

        - I am apprehensive about the gynecological examination for fear of pain
        - I am afraid that something abnormal will be discovered
        - I am apprehensive about consulting because I have already had a bad experience during a gynecological consultation
        - I have other more important health problems
        - I don't feel the need
        - Other (specify): ……………………………………………………….

PART 4: REGARDING THE SCREENING PAP SMEAR

- Are you vaccinated against HPV papillomavirus (vaccination started in 2008 for girls between 11 and 19 years old, before first sexual intercourse)
    - Yes, I had 2 or more doses
    - Yes, I had 1 dose
    - No
    - If no, why:
        - The vaccine did not exist when I was of age to be vaccinated
        - I did not know about this vaccine when I was of age to be vaccinated
        - I had concerns about the safety of the vaccine
        - I think I have a contraindication to the vaccine due to my disease or treatment
        - Other……………………………………………………………………
- At what age did you have your first sexual intercourse?
    - Before 15 years old
    - Between 15 and 19 years old
    - After 19 years old
    - I have never had sexual intercourse
- How many sexual partners have you had in your lifetime?
    - 0
    - 1
    - 2
    - More than 2
- Have you ever had a sexually transmitted infection in your lifetime (gonorrhea, chlamydia, genital herpes)?
    - Yes
    - No
- In your opinion, what is the purpose of the cervical Pap smear?
    - Evaluation of vaginal flora
    - Screening for sexually transmitted infections
    - Screening for cervical cancer
    - I don't know
    - Other (specify) ………………………………………………………….
- In your opinion, does the HPV papillomavirus vaccine exempt you from performing screening Pap smears?
    - Yes
    - No
    - I don't know
- In your opinion, how often should you have your cervical cancer screening Pap smear?
    - Every year
    - Every 2 years
    - Every 3 years
    - Every 5 years
    - I don't know
- In your opinion, when was your last cervical cancer screening Pap smear (cytology or HPV test)?
    - Less than 1 year ago

  - Between 1-3 years ago
  - Between 3-5 years ago
  - Between 5-10 years ago
  - I have never had a screening Pap smear
  - I don't know
- Has your rheumatologist, internist, or gynecologist talked to you about the risks related to papillomavirus, due to your disease/treatments?
  - Yes, there is no risk in my case
  - Yes, I know the risks in my case
  - Yes, but insufficiently, and I know the risks poorly if they exist
  - No
- Have you ever had an abnormal Pap smear or positive HPV test?
  - Yes
  - No
  - I don't know
  - If yes, specify the results:
    - ASCUS
    - LSIL
    - ASC-H
    - HSIL
    - AGC
    - HPV test +
- Have you ever had a colposcopy (examination of the cervix under a microscope)?
  - Yes
  - No
  - If yes, did you have biopsies?
    - Yes
    - No
  - If yes, what were the results of the biopsy?
    - Normal
    - Low-grade lesions (CIN 1)
    - High-grade lesions (CIN2, CIN 3, carcinoma in situ)
    - I don't know
  - What therapeutic approach was recommended?
    - Surveillance
    - Destruction (laser, cryotherapy)
    - Surgery (conization)
