# Supplementary material for: Cervical cancer screening practices among patients with autoimmune and inflammatory rheumatic diseases: a descriptive cross-sectional observational study
Source: BMC Womens Health. 2026 Apr 25;26:362. doi: 10.1186/s12905-026-04475-2 (PMC13371678; doi:10.1186/s12905-026-04475-2)
Supplement: Supplementary file 2 — Supplementary Material 2. [file 12905_2026_4475_MOESM2_ESM.pdf]

Supplemental Figure 2 : Cumulative incidence rate of CCS

A : Cumulative incidence rate of CCS frequency according to cervical risk

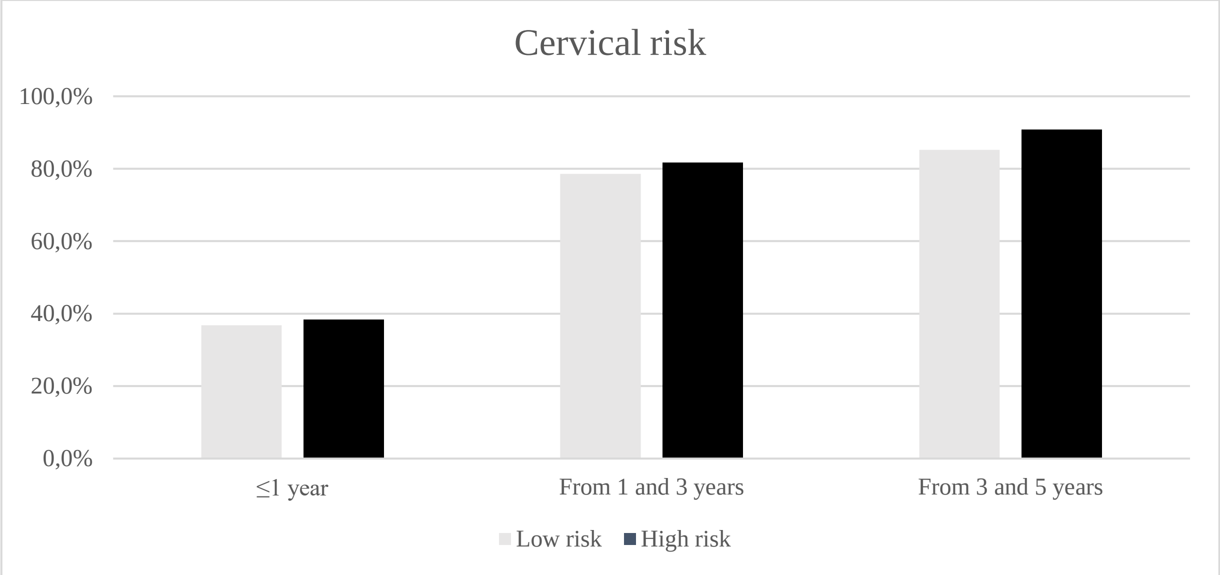

B : Cumulative incidence rate of CCS frequency according to disease

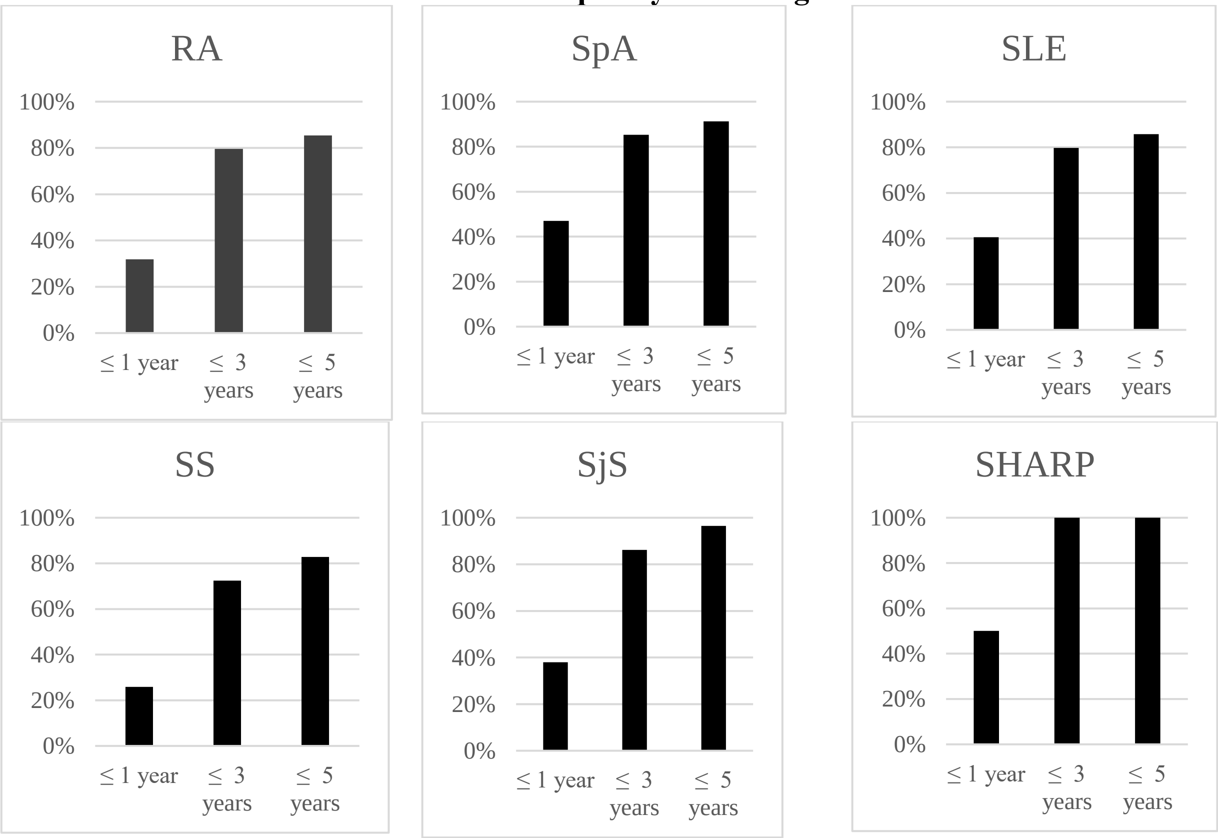

Low risk : *Women diagnosed with AIIRD and using csDMARD or no treatment.*
High risk : *Women diagnosed with AIIID and using bDMARD, tsDMARD or immunosuppressive therapy*
CCS : cervical cancer screening; AIIRD : autoimmune and inflammatory rheumatic diseases; RA : rheumatoid arthritis; PsA : spondyloartritis; SLE : systemic lupus erythematosus; SS : systemic sclerosis; SjS : sjogren's syndrome; bDMARD/csDMARD/tsDMARD : biological/conventional synthetic/targeted synthetic disease-modifying antiheumatic drug respectively
